# Role of a fluid-restrictive strategy in flap-surgery: A single center retrospective cohort study

**DOI:** 10.1097/MD.0000000000033673

**Published:** 2023-05-12

**Authors:** Harin Rhee, Ho Yoon Jeong, Changryul Claud Yi, Joo Hyoung Kim

**Affiliations:** a Department of Internal Medicine, Pusan National University School of Medicine, Yangsan, Republic of Korea; b Division of Nephrology, Biomedical Research Institute, Pusan National University Hospital, Pusan, Republic of Korea; c Division of Plastic Surgery, Biomedical Research Institute, Pusan National University Hospital, Pusan, Republic of Korea.

**Keywords:** acute kidney injury, flap surgery, fluid overload, surgical outcome

## Abstract

In this study, we evaluated the outcomes of flap surgery and the incidence of acute kidney injury (AKI) in patients who underwent flap surgery using a fluid-restrictive strategy. We retrospectively reviewed the consecutively collected medical records of patients who underwent flap surgery using the fluid-restrictive strategy of our hospital. The patients were divided into 2 groups based on the period of flap surgery: 2011 to 2014 (initiation period of the fluid-restrictive strategy) and 2015 to 2020 (implementation period). Outcomes of flap surgery and the incidence of AKI were evaluated based on percentage changes in cumulative fluid balance to initial body weight (%FO) on post-operative day 7. A total of 140 patients were enrolled in the study; 50 (35.7%) underwent flap surgery in 2011 to 2014 and 90 (64.3%) in 2015 to 2020. In 2015 to 2020, the median %FO significantly decreased from 2.7 (interquartile range [IQR]: 0.8–7.1) to 0.1 (IQR: −2.2 to 3.4%, *P* < .001), whereas the success rate significantly increased from 53.3% to 70.5% (*P* = .048) compared to 2011 to 2014. The incidence of AKI remained unchanged. In multivariate analysis, the odds ratio for success was 2.759 (95% confidence interval: 1.140–6.679) in 2015 to 2020 compared to 2011 to 2014. After successfully implementing the fluid-restrictive strategy, the success rate of flap surgery significantly increased without any further increase in the incidence of AKI. Our experience could serve as a model for implementing a fluid-restrictive strategy in flap surgery.

## 1. Introduction

Microvascular flap surgery is essential for patients requiring difficult soft tissue resections and having complex defects. Successful flap surgery is associated with an improved quality of life.^[1–3]^ For successful flap surgery, maintaining hyperdynamic systemic circulation and avoiding vasoconstrictors are crucial to ensure adequate flap perfusion.^[4,5]^ However, recent studies have revealed that administering a peri-operative vasopressor is not detrimental to free flap survival, as previously feared,^[6]^ and aggressive fluid delivery is associated with worse medical and surgical complications.^[7–9]^

Venous congestion in microvascular free flaps may increase the incidence of fat necrosis and wound complications, even when subclinical.^[7–9]^ Surgical reconstruction techniques can improve flap outcomes and reduce complications, but various other factors must be considered. Even a subtle vascular accident can lead to reoperation or post-operative scarring; attention should be paid to risk factors for these outcomes and a multidisciplinary strategy is necessary.

Our center initiated a fluid-restrictive strategy during flap surgery in 2011, which became a standard of care in 2015. We compared surgical and medical outcomes before and after implementing the fluid-restrictive strategy.

## 2. Methods

### 2.1. Patient selection

We retrospectively reviewed the consecutively collected data of those patients who underwent microvascular flap surgery in our hospital from January 2011 to December 2020. Patients with no input or output data during the hospital stay were excluded from the analyses. We divided the patients into those who underwent flap surgery between 2011 and 2014, when the fluid-restrictive strategy was started, and those who underwent surgery between 2015 and 2020, when the strategy became standard care. The Pusan National University IRB committee approved the study protocol [2110-014-108], and waived the requirement for informed consent.

### 2.2. Fluid-restrictive strategies during flap surgery

We monitored input and output from the day of surgery until post-operative day (POD) 7 or hospital discharge, whichever came first. We aimed to maintain the fluid balance between 0 and 500 mL per day. To reach this goal, we regulated the fluid infusion rate and used intravenous diuretics in cases of excess fluid accumulation (>1000 mL/d). Vasopressor or anti-hypertensive medications were administered to maintain target systolic blood pressure, and dexamethasone was applied to reduce flap edema.

To manage chronic kidney disease (CKD) patients during plastic surgery, we avoided nephrotoxic agents such as non-steroidal anti-inflammatory drugs and proton pump inhibitors, and adjusted antibiotic dosing according to the estimated glomerular filtration rate (eGFR). We serially monitored serum creatinine levels, until at least 3 days after the operation. In cases where acute kidney injury (AKI) was detected, the nephrology department was consulted for management.

### 2.3. Variables and definitions

We extracted patient data from medical charts including demographic (in turn obtained from nursing documentation) and comorbidity information (from physicians’ notes). Flap surgery-related details included flap location, use of vasopressors and diuretics during hospital stay, and flap outcomes. Daily input and output data were extracted from the electronic medical records, and we calculated percentage changes in cumulative fluid balance (CFB) relative to initial body weight.^[10]^ Percent fluid overload (%FO) was defined as changes in CFB at POD 7, which was calculated using the following formula: [(total fluid input until POD 7 or hospital discharge [L] − total output until POD 7 or hospital discharge [L])/body weight at admission [kg]] × 100.

### 2.4. Outcomes

The primary outcome was flap survival, and it was classified as success (no need for additional procedures such as debridement or skin graft to repair the remnant raw surface), partial necrosis (<50% necrosis of the flap), or total necrosis (>50% necrosis of the flap).

Secondary outcomes were the need for re-exploration surgery and incidence of AKI, which was defined as an increase in serum creatinine level ≥0.3 mg/dL from baseline within 48 hours or urine output <0.5 mL/kg/h for >6 hours.^[11]^

### 2.5. Statistical analysis

The normality of the data was verified using the Kolmogorov–Smirnov test. Continuous variables are expressed as the median and interquartile range (IQR) or mean ± standard deviation, as appropriate. Differences in parameters were compared using Student *t* test. Categorical variables are expressed as percentages, and differences in proportions were compared using the chi-square test. Factors predictive of flap outcome were analyzed using univariable or multivariable binary regression analysis. The final model was determined using backward model selection based on the Wald test, with a 0.2 threshold for including other predictors. All statistical tests were two-sided, and a *P* value < .05 was considered significant. We analyzed the data with IBM SPSS software (ver. 28.0; SPSS Inc., Chicago, IL).

## 3. Results

**Figure 1.**
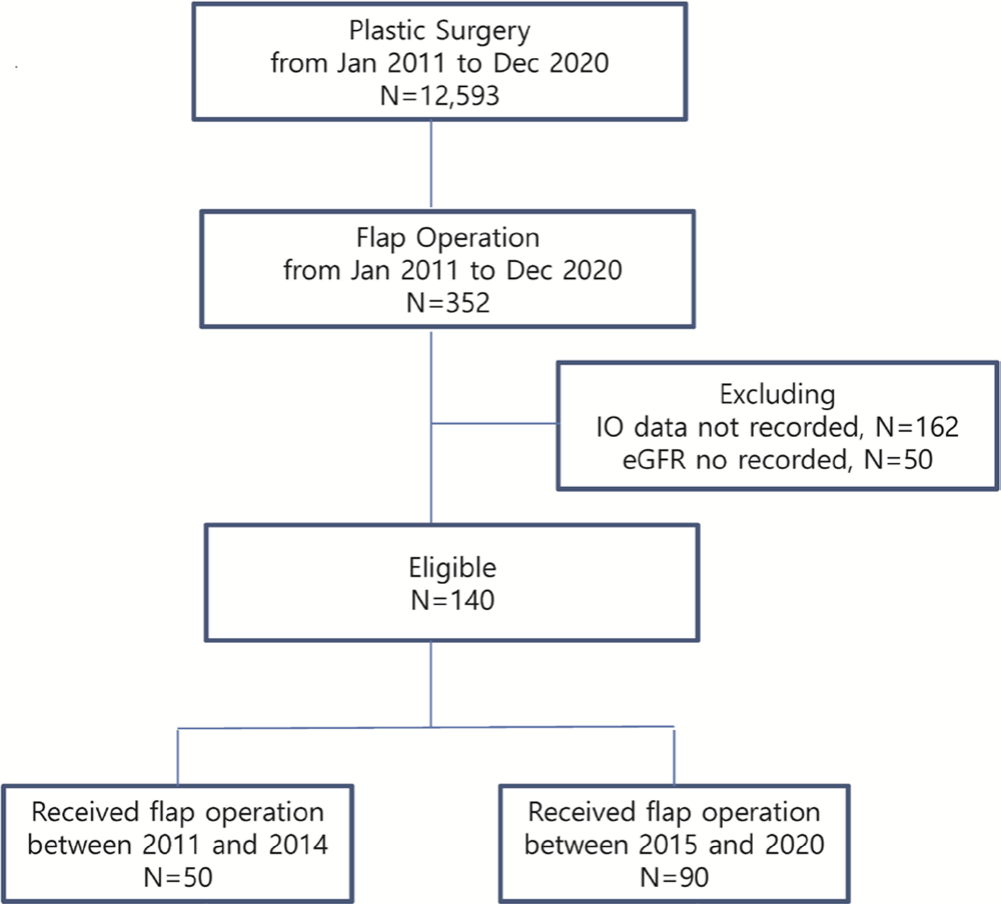
Flowchart of the study. eGFR = estimated glomerular filtration rate.

We performed 12,593 plastic surgeries from January 2011 to December 2020, and 352 (2.8%) were microvascular flap surgeries. Excluding the 162 cases without input/output records and 50 cases without eGFR data, we included 140 cases in this study. Among these cases, 50 (35.7%) underwent flap surgery in the period 2011 to 2014, while 90 (64.3%) underwent the surgery in 2015 to 2020 (Fig. 1).

### 3.1. Baseline characteristics

The mean age of the patients was 63.2 ± 13.7 years, 95 (67.9%) were male, and the mean body mass index was 22.9 ± 3.9 kg/m^2^. Most flap surgeries (83.1%) were performed on head and neck cancer patients. Cancer (84.3%) was the most prevalent comorbidity, and 16.4% of these patients received pre-operative radiation therapy. Hypertension (42.1%) was the second most common comorbidity, followed by CKD (7.1%) (Table 1).

**Table 1:** Baseline characteristics.

	Total	2011–2014	2015–2020	*P* value
	N = 140	N = 50	N = 90	
Demographics				
Age, yr	63.2 ± 13.7	59.7 ± 13.5	65.1 ± 13.5	.027
Male, %	67.9	68.0	67.8	.978
BMI, kg/m^2^	22.9 ± 3.9	22.4 ± 4.1	23.1 ± 3.9	.331
Smoker, %	34.3	36.0	33.3	.875
Causes of surgery				
Head & Neck	83.1	75.0	87.8	.127
Lower extremity	12.3	20.8	7.3	
Breast and trunk	3.8	4.2	3.7	
Upper extremity	12.3	20.8	7.3	
Comorbidities				
Diabetes, %	26.4	22.0	28.9	.376
Hypertension, %	42.1	38.0	44.4	.459
CKD, %	7.1	10.0	5.6	.328
CVD, %	8.6	8.0	8.9	.857
Heart ds, %	6.4	8.0	5.6	.572
Liver ds, %	2.9	0	4.4	.130
Lung ds, %	15.7	8.0	20.0	.062
Cancer	84.3	74.0	90.0	.013
PreOP treatment				
PreOP CTx	17.2	27.0	12.7	.056
PreOP RTx	16.4	22.2	13.8	.254
Laboratory findings				
Hb, g/dL	12.1 ± 1.5	12.0 ± 1.5	12.1 ± 1.5	.632
Platelet, 10 E3/µL	217.6 ± 83.8	219.5 ± 86.9	212.7 ± 71.7	.660
Basal eGFR	80.1 ± 30.1	81.4 ± 34.9	79.5 ± 27.8	.745

BMI = body mass index, CTx = chemotherapy, CVD = cardiovascular disease, eGFR = estimated glomerular filtration rate, OP = operation, RTx = radiation therapy.

**Figure 2.**
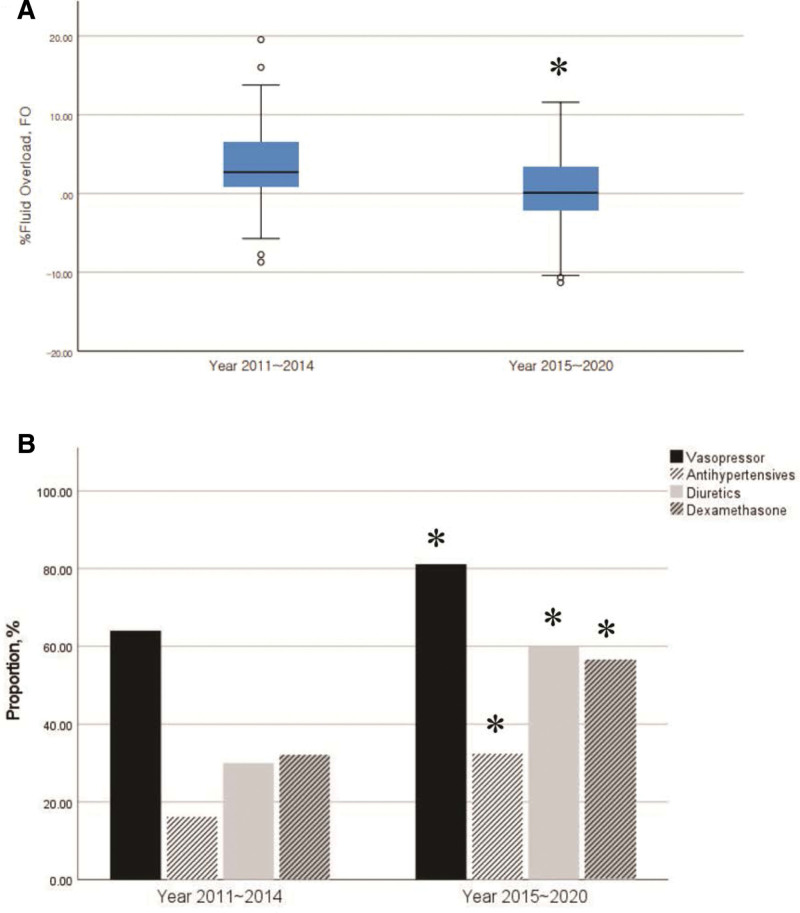
Percent fluid overload (A) and proportion of medication usage (B) changes between 2011–2014 and 2015–2020. * *P* < .05.

Following implementation of the fluid-restrictive strategy, there was a significant decrease in %FO from 2.7 (IQR: 0.8–7.1) in 2011 to 2014 to 0.1 (IQR: −2.2 to 3.4, *P* < .001, Fig. 2) in 2015 to 2020 (Fig. 2A). In addition, the use of vasopressors, diuretics, anti-hypertensives, and dexamethasone significantly increased after implementing the strategy (Fig. 2B).

### 3.2. Flap outcomes before and after fluid-restrictive strategy

**Figure 3.**
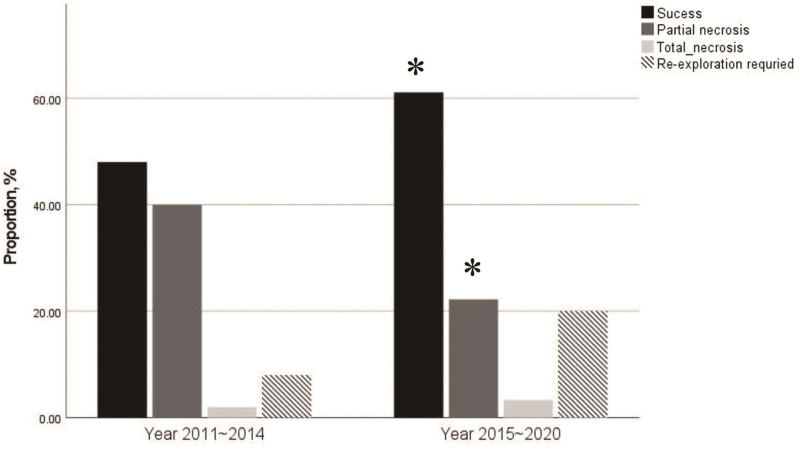
Changes in flap outcomes between 2011–2014 and 2015–2020. * *P* < .05.

Flap outcome was recorded in 123 (87.9%) out of 140 patients. The success rate increased from 53.3% in the period 2011 to 2014 to 70.5% in 2015 to 2020 (*P* = .048). The incidence of partial necrosis significantly decreased from 44.4% to 25.6% (*P* = .032) in the same period. Total necrosis was observed in 4 (3.3%) patients, and its incidence did not differ between the 2 periods. Twenty-two patients (15.7%) required re-exploration, which showed an increasing trend after implementing the fluid-restrictive strategy (Fig. 3). Detailed information on feeding vessels is summarized in Table S1, Supplemental Digital Content, http://links.lww.com/MD/I911.

In the multivariable binary regression analysis, the odds ratio (OR) for success was higher in the period 2015 to 2020 (OR: 2.759, 95% confidence interval [CI]: 1.140–6.679, *P* = .024) compared to 2011 to 2014 after adjusting for age, baseline eGFR, and %FO (Table 2). In addition, the period 2015 to 2020 was not associated with an increased risk of re-exploration (OR: 2.875, 95% CI: 0.915–9.033) in univariable and multivariable models.

**Table 2:** Role of fluid restrictive strategy on successful flap outcome and re-exploration requirement.

	OR (95% CI)	*P* value
Successful flap outcome		
Year 2015–2020	2.092 (0.977, 4.481)	.057
+ Age	2.035 (0.932, 4.441)	.074
+ Age, baseline eGFR	2.300 (1.027, 5.151)	.043
+ Age, baseline eGFR, %FO	2.759 (1.140, 6.679)	.024

%FO = percent fluid overload, eGFR = estimated glomerular filtration rate.

### 3.3. AKI incidence before and after fluid-restrictive strategy

AKI was observed in 35 (25.2%) of 139 patients. Of the 35 patients with AKI, 27 (77.1%) were stage 1, 6 (17.1%) were stage 2, and 2 (5.7%) were stage 3. The incidence of AKI did not significantly differ between 2011 to 2014 and 2015 to 2020, with 16 (32.0%) out of 50 patients and 19 (21.3%) out of 89 patients, respectively (*P* = .165). Univariable and multivariable analysis revealed that the period 2015 to 2020 was not a risk factor for AKI (OR: 0.577, 95% CI: 0.264–1.260). Significant predictors of AKI after flap surgery were baseline lower eGFR (OR: 0.950, 95% CI: 0.932–0.9680) and %FO (OR: 1.142, 95% CI: 1.038–1.255) (Table 3).

**Table 3:** Factors associated with AKI after flap surgery.

	HR (95% CI)	*P* value
Baseline eGFR	0.950 (0.932, 0.968)	<.001
%FO	1.142 (1.038, 1.255)	.006

Factors were adjusted with age, baseline estimated glomerular filtration rate (eGFR), use of angiotensin receptor blocker, and % fluid overload (%FO).

AKI = acute kidney injury.

## 4. Discussion

This study aimed to evaluate the outcomes of a fluid-restrictive strategy in patients undergoing flap surgery. The strategy was initiated in 2011 and became a standard of care in 2015. Compared to 2011 to 2014, the frequency of vasopressor and diuretic administration increased, whereas the %FO significantly decreased during 2015 to 2020. In addition, the success rate of flap surgery increased, and the incidence of partial necrosis decreased, whereas the need for re-exploration surgery and the incidence of AKI remained unchanged. After adjusting for age, eGFR, and %FO level, the odds of success increased 2.8-fold in 2015 to 2020 compared to 2011 to 2014.

Massive fluid infusion was traditionally recommended for flap surgery, as it was believed to ensure adequate perfusion of the flap and help avoid the need for vasopressors.^[4]^ However, excessive fluid administration during flap surgery is a risk factor for poor outcomes,^[4,12]^ where edema in the flap or recipient can result in major complications. Based on a literature review of 84 articles on perioperative fluid management, Brinkman et al^[13]^ recommended that maintenance fluid should not exceed 6 mL/kg/h. Karamanos et al^[14]^ found that restrictive fluid resuscitation (<7 mL/kg/h) can increase blood flow to the flap and reduce wound-related complications in breast reconstruction patients. Funk et al^[15]^ administered goal-directed fluid therapy to patients undergoing microvascular free flap reconstruction after mastectomy, aiming to maintain stroke volume variation at 13% or less; they reported that intra-arterial blood pressure monitoring was helpful for achieving this goal, and the intervention group had a baseline fluid infusion rate of 5 mL/kg/h.

The novelty of our strategy was that we focused on net fluid balance rather than the fluid infusion rate alone, by regulating the fluid infusion rate and intravenous diuretics. The median %FO between 2015 and 2020 was nearly 0, indicating no extra fluid accumulation at POD 7. This approach was expected to be more effective in CKD patients who have difficulty regulating fluid balance.^[16]^ Inability of the kidneys to eliminate excess salt and water results in an expansion of the extracellular water compartment, which promotes tissue edema.^[17]^ Of the 140 patients in this study, 10 (7.1%) had CKD; the median eGFR of these patients was 20.4 (IQR: 8.8–48.7 mL/min/1.73 m^2^) and the median %FO was −0.4 (IQR: −4.5 to 3.0), which were not significantly different from those of the patients without CKD. The flap outcome was successful in 7 out of 10 CKD patients, and the remaining 3 patients experienced partial necrosis. There were no instances of total necrosis in the CKD group. These findings suggest a need for further prospective studies to investigate the influence of zero-fluid balance on flap outcomes in CKD patients.

Fluid excess of > 10% of body weight in critically ill patients has been associated with respiratory failure, the need for a mechanical ventilator, and an increased risk of sepsis.^[18]^ However, no established guidelines define an acceptable %FO range for plastic surgery patients. Due to the limited number of patients in our study, we could not conduct further analysis to determine the cutoff level of the target %FO that predicts a successful flap outcome. Future studies with more patients are necessary to address this issue.

AKI was observed in 25.2% of 139 of our patients; 77.1% were stage 1, and 2 (5.7%) were stage 3. Prowle et al^[19]^ reported that the incidence of post-operative AKI after non-cardiac major surgery ranges from 5% to 40%, where the rate differed by surgery type. Although plastic surgery is a non-emergent, non-major surgery, the incidence of AKI is comparable to that of major surgery. Considering the higher prevalence of stage 1 than stage 3 AKI, it is important to compare long-term outcomes to those of major surgeries. After adjusting for age, baseline eGFR, and ARBs, an increase in %FO was found to be an independent risk factor for the development of AKI in patients underwent flap surgery, consistent with previous reports.^[20,21]^

Our study had several strengths. First, we calculated the increase in CFB relative to the initial body weight, allowing for individualized fluid balance measurement independent of baseline kidney function and initial body weight. Second, our study demonstrated the importance of a multidisciplinary approach to flap surgery.

However, our study also had some limitations. First, it was a single-center, retrospective study that included a relatively small number of patients. Nevertheless, the consistency of our results with previous findings confirms the beneficial effect of a fluid-restrictive strategy in flap surgery. The improvement in outcomes of flap surgery from 2015 to 2020 seems to be attributed to the fluid-restrictive strategy, whereas unmeasured factors, such as improvement in surgeon skills with time, may be potential sources of bias.

## 5. Conclusion

Our center adapted a fluid-restrictive strategy in 2011, which became the standard of care in flap surgery by 2015. Compared to 2011 to 2014, the success rate of flap surgery significantly increased in 2015 to 2020, without a corresponding increase in the need for re-exploration operations or AKI incidence. Our experience could serve as a model fluid-restrictive strategy for flap surgery.

## Author contributions

**Conceptualization:** Harin Rhee.

**Investigation:** Ho Yoon Jeong, Changryul Claud Yi.

**Methodology:** Harin Rhee.

**Supervision:** Joo Hyoung Kim.

**Writing – original draft:** Harin Rhee.

**Writing – review & editing:** Harin Rhee.

## Supplementary Material



## References

[1] Lahtinen S Koivunen P Ala-Kokko T. Quality of life after free flap surgery for cancer of the head and neck in patients with or without postoperative complications. Eur Arch Otorhinolaryngol. 2018;275:2575–84. 30143854 10.1007/s00405-018-5103-4

[2] Lofstrand J Nyberg M Karlsson T. Quality of life after free fibula flap reconstruction of segmental mandibular defects. J Reconstr Microsurg. 2018;34:108–20. 28905342 10.1055/s-0037-1606537

[3] Melan JB Philouze P Pradat P. Functional outcomes of soft palate free flap reconstruction following oropharyngeal cancer surgery. Eur J Surg Oncol. 2021;47:2265–71. 33994058 10.1016/j.ejso.2021.04.044

[4] Zheng G Liu J Yu P. Intraoperative fluid management implies insignificant influence to surgical outcomes in head and neck microvascular reconstruction cases. Plast Reconstr Surg. 2021;147:627e–33e. 10.1097/PRS.0000000000007777 33776038

[5] Massey MF Gupta DK. The effects of systemic phenylephrine and epinephrine on pedicle artery and microvascular perfusion in a pig model of myoadipocutaneous rotational flaps. Plast Reconstr Surg. 2007;120:1289–99. 17898602 10.1097/01.prs.0000279371.63439.8d

[6] Goh CSL Ng MJM Song DH. Perioperative vasopressor use in free flap surgery: a systematic review and meta-analysis. J Reconstr Microsurg. 2019;35:529–40. 31042803 10.1055/s-0039-1687914

[7] Ettinger KS Arce K Lohse CM. Higher perioperative fluid administration is associated with increased rates of complications following head and neck microvascular reconstruction with fibular free flaps. Microsurgery. 2017;37:128–36. 27098099 10.1002/micr.30061

[8] Burkhard JP Pfister J Giger R. Perioperative predictors of early surgical revision and flap-related complications after microvascular free tissue transfer in head and neck reconstructions: a retrospective observational series. Clin Oral Investig. 2021;25:5541–50. 10.1007/s00784-021-03864-1 PMC8370926 33686470

[9] Booi DI. Perioperative fluid overload increases anastomosis thrombosis in the free TRAM flap used for breast reconstruction. Eur J Plast Surg. 2011;34:81–6. 21475651 10.1007/s00238-010-0466-9 PMC3062757

[10] Bouchard J Soroko SB Chertow GM.; Program to Improve Care in Acute Renal Disease (PICARD) Study Group. Fluid accumulation, survival and recovery of kidney function in critically ill patients with acute kidney injury. Kidney Int. 2009;76:422–7. 19436332 10.1038/ki.2009.159

[11] Section 2: AKI definition. Kidney Int Suppl (2011). 2012;2:19–36. 25018918 10.1038/kisup.2011.32 PMC4089595

[12] Haughey BH Wilson E Kluwe L. Free flap reconstruction of the head and neck: analysis of 241 cases. Otolaryngol Head Neck Surg. 2001;125:10–7. 11458207 10.1067/mhn.2001.116788

[13] Brinkman JN Derks LH Klimek M. Perioperative fluid management and use of vasoactive and antithrombotic agents in free flap surgery: a literature review and clinical recommendations. J Reconstr Microsurg. 2013;29:357–66. 23599215 10.1055/s-0033-1343955

[14] Karamanos E Walker R Wang HT. Perioperative fluid resuscitation in free flap breast reconstruction: when is enough enough? Plast Reconstr Surg Glob Open. 2020;8:e2662. 32537330 10.1097/GOX.0000000000002662 PMC7253255

[15] Funk D Bohn J Mutch W. Goal-directed fluid therapy for microvascular free flap reconstruction following mastectomy: a pilot study. Plast Surg (Oakv). 2015;23:231–4. 26665136 10.4172/plastic-surgery.1000937 PMC4664136

[16] Mitsides N Alsehli FMS Mc Hough D. Salt and water retention is associated with microinflammation and endothelial injury in chronic kidney disease. Nephron. 2019;143:234–42. 31514183 10.1159/000502011

[17] Mitra S. Extracellular hydration, cardiovascular risk, and the interstitium: a three-dimensional view. Kidney Int. 2014;85:510–2. 24583986 10.1038/ki.2013.481

[18] Claure-Del Granado R Mehta RL. Fluid overload in the ICU: evaluation and management. BMC Nephrol. 2016;17:109. 27484681 10.1186/s12882-016-0323-6 PMC4970195

[19] Prowle JR Forni LG Bell M. Postoperative acute kidney injury in adult non-cardiac surgery: joint consensus report of the acute disease quality initiative and perioperative quality initiative. Nat Rev Nephrol. 2021;17:605–18. 33976395 10.1038/s41581-021-00418-2 PMC8367817

[20] Yerram P Karuparthi PR Misra M. Fluid overload and acute kidney injury. Hemodial Int. 2010;14:348–54. 20955269 10.1111/j.1542-4758.2010.00498.x

[21] Prowle JR Echeverri JE Ligabo EV. Fluid balance and acute kidney injury. Nat Rev Nephrol. 2010;6:107–15. 20027192 10.1038/nrneph.2009.213

