# Supplementary material for: Role of a fluid-restrictive strategy in flap-surgery: A single center retrospective cohort study
Source: Medicine (Baltimore). 2023 May 12;102(19):e33673. doi: 10.1097/MD.0000000000033673 (PMC10174412; doi:10.1097/MD.0000000000033673)
Supplement: Supplementary file 1 [file medi-102-e33673-s001.pdf]

Supplement Table 1. Detailed information on Flap outcomes

	Total<br>N=140	2011~2014<br>N=50	2015~2020<br>N=90	P-value
Success, N(%)	79(64.2)	24(53.3)	55(70.5)	0.048
Partial Necrosis, %	40(32.5)	20(44.4)	20(25.6)	0.023
Total Necrosis, %	4(3.3)	1(2.2)	3(3.8)	0.625
Required re-exploration, N(%)	22(15.7)	4(8.0)	18(20.0)	0.062
Arterial problem	5(3.6)	1(2.0)	4(4.4)	0.366
Venous problem	7(5.0)	1(2.0)	6(6.7)	
Both artery + venous	3(2.1)	0	3(3.3)	
Non-vascular problem	7(5.0)	2(4.0)	5(5.6)	
